# Abundantly Present miRNAs in Milk-Derived Extracellular Vesicles Are Conserved Between Mammals

**DOI:** 10.3389/fnut.2018.00081

**Published:** 2018-09-18

**Authors:** Martijn J. C. van Herwijnen, Tom A. P. Driedonks, Basten L. Snoek, A. M. Theresa Kroon, Marije Kleinjan, Ruurd Jorritsma, Corné M. J. Pieterse, Esther N. M. Nolte-‘t Hoen, Marca H. M. Wauben

**Affiliations:** ^1^Department of Biochemistry and Cell Biology, Faculty of Veterinary Medicine Utrecht University, Utrecht, Netherlands; ^2^Theoretical Biology and Bioinformatics, Department of Biology, Science4Life Utrecht University, Utrecht, Netherlands; ^3^Department of Farm Animal Health, Faculty of Veterinary Medicine Utrecht University, Utrecht, Netherlands; ^4^Plant-Microbe Interactions, Department of Biology, Science4Life Utrecht University, Utrecht, Netherlands

**Keywords:** milk, miRNA, extracellular vesicles, exosomes, immune modulation

## Abstract

Mammalian milk is not only a source of nutrition for the newborn, but also contains various components that regulate further development. For instance, milk is an abundant source of microRNAs (miRNAs), which are evolutionary conserved small non-coding RNAs that are involved in post-transcriptional regulation of target mRNA. MiRNAs present in milk can occur in extracellular vesicles (EVs), which are nanosized membrane vesicles released by many cell types as a means of intercellular communication. The membrane of EVs protects enclosed miRNAs from degradation and harbors molecules that allow specific targeting to recipient cells. Although several studies have investigated the miRNA content in milk EVs from individual species, little is known about the evolutionary conserved nature of EV-associated miRNAs among different species. In this study, we profiled the miRNA content of purified EVs from human and porcine milk. These data were compared to published studies on EVs from human, cow, porcine, and panda milk to assess the overlap in the top 20 most abundant miRNAs. Interestingly, several abundant miRNAs were shared between species (e.g., let-7 family members let-7a, let-7b, let-7f, and miR-148a). Moreover, these miRNAs have been implicated in immune-related functions and regulation of cell growth and signal transduction. The conservation of these miRNA among species, not only in their sequence homology, but also in their incorporation in milk EVs of several species, suggests that they are evolutionarily selected to regulate cell function in the newborn.

## Introduction

Mother's milk provides the newborn with more than only nutrition: it contains various complex macromolecular structures that carry signaling molecules that are delivered to the newborn. One of these signaling moieties are extracellular vesicles (EVs). EVs are cell-derived lipid bilayer-enclosed vesicles containing selectively incorporated proteins, lipids, and nucleic acids (mainly small RNAs) (1–3), which are in turn selectively delivered to recipient cells to modulate their functions (4, 5). Hence, the transfer of maternal milk EVs to the newborn allows for cross-organism communication.

EVs are generally classified into exosomes and microvesicles, which have distinct biogenesis pathways and may differ in size and cargo (1, 2). Currently available methodologies do not allow discrimination between exosomes and microvesicles as the size, surface markers, and buoyant densities of these vesicle subtypes overlap (2). Therefore, we will use the generic term EVs in this study even though previously published studies attribute their findings to certain subtypes, mostly exosomes.

EVs have been identified and characterized in human milk (6–9), as well as in milk from other species, such as cow (10, 11), buffalo (12), pig (13), wallaby (14), horse (15), camel (16), rat (17), and panda (18). Additionally, extensive miRNA profiling of milk EVs has been performed in human (8–20), cow (21), pig (13, 22), and panda (18), and several high abundant miRNAs have been identified in each of these species. These abundantly miRNA species might be involved in the specific targeting of signaling pathways in the newborn. However, the EV isolation procedures used in these studies only enabled enrichment for milk EVs but precluded isolation of pure EVs (23). In the current study, we employed an optimized protocol that we previously developed for recovery and characterization of EVs from fresh milk (7, 9) in order to assess which miRNAs were most abundant in purified EVs from human and porcine milk. Additionally, we compared the identified miRNA profiles from this study with the previously reported high abundant EV-associated miRNAs from human, bovine, porcine, and panda milk. This allowed us to compare the impact of EV isolation procedures on the analysis of miRNAs in milk EVs and to examine the conserved nature of highly abundant miRNAs present in milk EVs from different species. Furthermore, we speculate on the potential impact on the newborn's development via these conserved milk EVs-associated miRNAs.

## Methods

### Milk EVs isolation

Milk EVs were isolated as previously described (7, 9). Fresh human milk from a pool of four breast-feeding mothers 3 until 9 months after delivery was obtained after informed consent of the donors and approval by the local ethics committee. Porcine milk was obtained from two sows between 2 and 3 weeks after delivery after approval by the local ethics committee. Raw milk was centrifuged twice at 3,000 × *g* (Beckman Coulter Allegra X-12R, Fullerton, CA) and the milk supernatant was subjected to differential centrifugation at 5,000 × *g* and 10,000 × *g* in sterilized and new SW40 tubes (Beckman Coulter). The 10,000 × *g* supernatant was loaded onto a sucrose gradient (ranging from 2.0 to 0.4 M sucrose) and ultracentrifuged at 192,000 × g (in a Beckman Coulter Optima L-90K with a SW40 rotor) for 15–18 h (k-factor 144.5). EV-containing fractions (1.12–1.18 g/ml) were harvested, pooled, and centrifuged at 100,000 × g for 65 min. After centrifugation, supernatant was removed and EV pellets were aliquoted and stored at 80°C.

### EV-RNA isolation

Small RNA was isolated using the miRNeasy Micro kit according to the small RNA enrichment protocol provided by the manufacturer (Qiagen, Hilden, Germany). RNA yield and size profile were assessed using Agilent 2100 Bioanalyzer and Pico 6000 RNA chips (Agilent Technologies, Waldbronn, Germany) (see Table 1 for comparison between studies).

**Table 1:** Overview of experimental details for isolation and characterization of milk EVs miRNAs in this study and previously published studies.

	**This study**	**Zhou (8)**	**Simpson (20)**	**Liao (19)**	**Izumi (21)**	**This study**	**Gu (13)**	**Chen (22)**	**Ma (18)**
Species	Human	Human	Human	Human	Bovine	Porcine	Porcine	Porcine	Panda
Donors	Pool of 4 individuals	4 Individuals	54 Individuals	12 Individuals	3 Individuals	2 Individuals	3 Individuals	NR	3 Individuals
Time post-partum	3–9 months	60 days	3 months	1–8 months	NR	3–4 weeks	0–28 days	1–5 days	0–30 days
Storage of whole milk at −80°C	No	Yes	Yes	Yes	Yes	No	No	Yes	No
EV isolation procedure	Differential centrifugation; density gradient	Differential centrifugation; Exoquick	Differential centrifugation; Exoquick	Exoquick	Differential centrifugation; ultra- centrifugation	Differential centrifugation; density gradient	Exoquick	Differential centrifugation; ultra- centrifugation	Differential centrifugation; Exoquick
miRNA extraction kit	miRNeasy	TRIzol-LS	miRNeasy	TRIzol	miRNeasy	miRNeasy	TRIzol-LS	TRIzol	TRIzol-LS
Library preparation kit	New England Biolabs, NebNext	NR	Epicentre Biotechnologies, ScriptMiner	Illumina TruSeq	Not applicable	Illumina TruSeq	NR	NR	NR
miRNA profiling method	HiSeq 2000 (Illumina)	Genome Analyzer II (Illumina)	HiSeq 2000 (Illumina)	HiSeq 2500 (Illumina)	Microarray (670 bovine miRNA)	HiSeq 2000 (Illumina)	Genome Analyzer II (Illumina)	Solexa sequencing	Genome Analyzer II (Illumina)

*To be selected, a study should apply a method for the enrichment of EVs from the milk sample in combination with miRNA profiling by microarray or small RNA sequencing. The selected papers focused on human, bovine, porcine or panda milk. Variations between sample numbers and time of sample collection are indicated, as well as differences in milk storage, EVs/miRNA isolation procedures, and miRNA profiling methods. NR, not reported*.

### Preparation of small RNA sequencing libraries

Eight nanograms of milk EV-derived small RNA was treated with DNase (Turbo DNA-free kit (Life Technologies, Carlsbad, CA), pelleted using Pellet Paint (Merck, Darmstadt, Germany), and subsequently reconstituted in 6 μl milliQ water (human samples) or 12 μl milliQ water (porcine samples). For the human samples, cDNA libraries were prepared using the NebNext small RNA library prep kit for Illumina (New England Biolabs, Ipswich, MA) with the following adaptations: 3′ adapter ligation was carried out overnight at 16°C; Kapa HiFi Readymix 2 × PCR mastermix (Kapa Biosystems, Wilmington, MA) was used for PCR amplification as follows: 2 min at 95°C; 17 cycles of 20 s at 98°C, 30 s at 62°C, 15 s at 70°C, and a final elongation step of 5 min at 70°C. For the porcine samples, cDNA libraries were prepared as indicated by manufacturer with the Illumina TruSeq kit (Illumina Inc., San Diego, CA) with PCR amplification up to 17 cycles. cDNA was purified using AMPure XP beads (Beckman Coulter, Brea, CA) and quantified using Agilent 2100 Bioanalyzer and DNA HiSensitivity chips. Adapter dimers were removed by TBE gel purification (146–400 nt of which 126 nt derive from adapters for human, and a size range of 150–175 bp for porcine samples).

### miRNA profiling

Data quality was checked with FastQC and reads were processed with cutadapt (v1.8) to remove low quality reads. Sequences with a minimal length of 15 bp (human) or maximum length of 25 bp (porcine) after adapter trimming were retained. The end-trimmed sequences were mapped to the miRNA hairpin sequences using bowtie (v1.1.1 for human and v1.2.2 for porcine) with default settings. miRNA sequences were retrieved from miRbase (v21) and for both human and porcine the combined number of reads per miRNA was used to find the top miRNAs. Supplementary File 1 lists all miRNAs identified in human and porcine milk EV, together with sequencing depth and mapping rates. Sequencing data were deposited in NCBI's Gene Expression Omnibus (GEO) under accession number GSE118409.

### Literature search previously published milk EVs miRNA analysis

To collect published data on miRNA profiling of milk EVs from different species, studies listed in PubMed up to March 2018 were selected based on the keywords “milk” and “microRNA(s),” “miRNA(s),” “extracellular vesicle(s),” “exosome(s),” or “microvesicles.” Additionally, the bibliographies from the retrieved articles were searched to find additional sources. Subsequently, the experimental procedures in these papers were evaluated. Only those papers in which extensive miRNA profiling was performed either by deep sequencing or microarray on milk samples enriched (by differential centrifugation and/or precipitation) for milk EVs were selected for further comparison. In total seven studies were selected to compare the most abundantly present miRNAs (see Table 1 for comparison of selected studies).

### *In silico* analysis

Tarbase (24) was used to screen for validated targets of identified miRNAs and TargetScan (25) was used to assess conservation of these target sites. Funrich (26) was used for GO analysis on the identified targets.

## Results

### miRNA profiling of purified EVs from human and porcine milk

We show for the first time the miRNA profile of purified milk EVs isolated after differential centrifugation followed by density gradient separation. We identified 309 mature miRNAs in human milk EVs and 218 mature miRNAs in porcine milk EVs (Supplementary File 1), These numbers are in range with the miRNAs that were previously identified in milk EV enriched samples of human [*n* = 602 (8), *n* = 125 (20) and *n* = 610 (19); average of 446 ± 278 miRNAs] and porcine [*n* = 234 (13) and *n* = 491 (22); average of 363 ± 182 miRNAs]. Hence, the number of identified miRNAs seems not to differ greatly between purified EVs and EV-enriched samples or the RNA extraction and profiling methods used.

### Milk EVs from different species share abundantly present miRNAs

In order to identify similarities in the miRNA composition of milk EVs from different species, we compared the top 20 most abundant miRNAs detected in purified milk EVs to the reported top 20 most abundant miRNAs in the selected publications. We observed substantial overlap in the top-ranked miRNAs observed in the different studies, with 19 miRNAs being abundantly detected in at least four out of nine studies (Table 2). Interestingly, four miRNAs were identified in high abundance in all four species examined. These included miRNA let-7 family members let-7a, let-7b, and let-7f, as well as miR-148a. These miRNAs were fully conserved at the sequence level in all species (Supplementary File 1). In addition to the similarities observed in miRNA content of EV, we also identified miRNAs that were abundantly present in milk EVs from all but one species. For example, miR-20a, miR-26a, and miR-141 were not present in the top 50 most abundant miRNAs in any of the three porcine studies, while these miRNAs are in the porcine genome. Additionally, let-7c was not abundantly present in any of the four human studies (see Supplementary File 1 for the full Table 2). Furthermore, we also observed differences in miRNA profiles reported in studies investigating milk EVs from the same species. Many factors may underlie these differences, including pre-analytic variables, differences in milk EVs and RNA isolation protocols, time in lactation period, and inter-individual differences in milk composition.

**Table 2:** Overlap in top 20 most abundant miRNAs detected in milk EVs from human, cow, pig, and panda.

**Top 20 ranked**	**This study**	**Zhou et al. (8)**	**Simpson et al. (20)**	**Liao et al. (19)**	**Izumi et al. (21)**	**This study**	**Gu et al. (13)**	**Chen et al. (22)**	**Ma et al. (18)**	**Times identified in top 20**
**miRNA**	**Human**	**Human**	**Human**	**Human**	**Bovine**	**Porcine**	**Porcine**	**Porcine**	**Panda**	
**let-7a-5p**	5	6	6	17	8	1	10	8	5	9
**miR-148a-3p**	2	1	1	2	14		1	•	2	7
miR-30a-5p	10	NR	13	11	•	2	2	13	8	7
**let-7f-5p**	9	3	7	•	17	6	NR	9	13	7
miR-30d-5p	1	NR	3	4	•	5	5	•	17	6
**let-7b-5p**	8	NR	4	15	4		NR	•	1	5
miR-21-5p	7	NR	10	•		4	12	•	15	5
miR-22-3p	18	NR	2	1		•	NR	17	16	5
miR-320a-3p	17	NR	•	20	15	10	NR	3	•	5
miR-191-5p	•	NR	•	13		3	9	7	•	4
miR-200a-3p	3	9	5	16	•		NR		•	4
miR-181a-5p	•	NR	•	3		20	NR	4	10	4
miR-92a-3p	•	NR	•	8		13	NR	14	3	4
miR-182-5p	•	8		10		19	4			4
miR-141-3p		7	20	5	10		NR		•	4
let-7g-5p	11	NR	•	14	•	9	NR	•	9	4
let-7c		NR			13	7	NR	10	19	4
miR-375-3p	14	NR	17	9	•		13			4
miR-26a-5p	•	NR	18	6	20		NR		20	4
miR-200c-3p	4	NR	•	•	9	8	NR		•	3

*The top 20 most abundant miRNA (this study) were compared to the top 20 miRNA reported in the indicated publications on milk EVs (see Supplementary File 1 for all data; Table shows the 20 most abundant miRNA ranked according to their presence in the top 20 of the selected studies). If a miRNA was present in the top 20, their ranking is stated and if their position is 21–50, this is depicted with •. NR, not reported (these two studies only reported the top 10 or the top 13 most abundant miRNA). Red shading indicates a high ranking (position 1–10), while blue indicates a low ranking (position 11-20). Those miRNA that were identified within the top 20 of all species (in at least one human or porcine study, and in the bovine and panda study) are depicted in bold*.

### Conserved milk EV-associated miRNAs have immune-related functions

Milk EVs have been indicated to modulate immune cells (6) and promote epithelial cell growth (27). Importantly, milk-derived miRNAs have been shown to survive harsh conditions including RNase digestion (8, 13, 14, 18), low pH (13, 14, 18), and *in vitro* gastro-intestinal digestion (19, 28). This indicates that milk EVs resist gastro-intestinal conditions and therefore presumably remain intact in the newborn gastro-intestinal tract. Even though no strong direct *in vivo* evidence exists, milk EVs can be taken up *in vitro* by intestinal epithelial cells (19, 29, 30), vascular epithelial cells (31), and macrophages (11, 21, 32). This suggests that EV cargo can reach the cells at the mucosa of the newborn.

In order to link the conserved milk EV miRNAs to relevant physiological processes, we identified 24 validated target (Supplementary File 1) and used GO analysis to determine that these targets are involved in “cell growth and/or maintenance,” “cell communication” and “signal transduction” (Supplementary File 1). In fact, let-7a/b/f-5p and miR-148-3p have been shown to regulate signal transduction by downregulating the transcription factor NF-κB resulting in a dampened immune response (33–35). Collectively, these data suggest that milk EVs harbor evolutionary miRNAs with immunomodulatory functions that can regulate the newborns development.

## Discussion

In this study, we isolated EVs from human and porcine milk and determined the presence and abundance of miRNAs. The top 20 of most abundantly present miRNAs from the purified human or porcine milk EVs were compared to previously reported miRNAs that were profiled from EV-enriched samples from human, bovine, porcine, and panda milk. Four specific miRNAs appeared to be highly abundant and conserved not only in their sequence, but also in their presence in milk of several species.

At first glance the number of isolated miRNA species does not seem to differ greatly between purified and EV-enriched milk samples and the four conserved miRNAs were identified irrespective of further EV purification and disparate RNA isolation and profiling methods. However, we also observed clear differences along the line reported earlier by van Deun et al. (36). For instance, miR-100-5p was only detected in high abundance in the purified human milk EVs from this study and was not present in the top 50 of other human studies. In contrast, miR-30b-5p was not abundantly present in purified human milk EV, while it was detected in the three other human studies. For porcine milk EVs there were many discrepancies between the identification of miRNAs between purified EVs compared to the other isolation methods. This could also be caused by a divergence in RNA isolation, library preparations, and sequencing method, which was different for this study compared to Gu et al. and Chen et al. A first step would be to standardize milk collection, milk storage, milk EV isolation, miRNA extraction, miRNA profiling, and data analysis. Finally, adequate reporting of experimental details should be a priority in the EV field to increase rigor and reproducibility (37).

In conclusion, the evolutionary conserved character of a selected set of miRNAs in milk EVs is remarkable. Their presence in EVs suggests a possible conserved role in maternal to newborn cross-organism communication. By targeting a variety of genes, including those that are involved in the regulation of the newborn's epithelial barrier and immune system these maternal milk EV-associated miRNAs might contribute to the guided further development of the newborn.

## Ethics statement

For human subjects: This study was carried out in accordance with the recommendations of Medical Research Involving Human Subjects Act (WMO), Medical Research Ethics Committee UMC Utrecht. The protocol was approved by the Medical Research Ethics Committee UMC Utrecht. All subjects gave written informed consent in accordance with the Declaration of Helsinki. For animal subjects: This study was carried out in accordance with the recommendations of guideline 2010/63/EU, dierexperimentencommissie (DEC) Utrecht, in which no approval for milk sampling under the described conditions was required.

## Author contributions

MH, RJ, CP, EH, and MW conceived and designed the study. MH, TD, BS, AK, and MK performed experiments and analyzed the data. MH, TD, BS, AK, EH, and MW wrote and revised the manuscript. All authors contributed to editing the paper.

### Conflict of interest statement

The authors declare that the research was conducted in the absence of any commercial or financial relationships that could be construed as a potential conflict of interest. The reviewer PC declared a past co-authorship with several of the authors EH & MW to the handling Editor.
